# A Symbiotic Approach to Generating Stress Tolerant Crops

**DOI:** 10.3390/microorganisms9050920

**Published:** 2021-04-25

**Authors:** Regina S. Redman, Yong Ok Kim, Sang Cho, Malia Mercer, Melissa Rienstra, Ryan Manglona, Taylor Biaggi, Xin-Gen Zhou, Martin Chilvers, Zachery Gray, Russell J. Rodriguez

**Affiliations:** 1 Adaptive Symbiotic Technologies, Seattle, WA 98105-5663, USA; yongokkim4@gmail.com (Y.O.K.); scho@adsymtech.com (S.C.); mmercer@adsymtech.com (M.M.); mrienstra@adsymtech.com (M.R.); rmanglona@adsymtech.com (R.M.); tbiaggi@adsymtech.com (T.B.); zgray@adsymtech.com (Z.G.); rjrodriguez@adsymtech.com (R.J.R.); 2 Texas A&M AgriLife Research Center, Beaumont, TX 77713, USA; xzhou@aesrg.tamu.edu; 3 Department of Plant, Soil and Microbial Sciences, Michigan State University, East Lansing, MI 48824, USA; chilvers@msu.edu

**Keywords:** symbiosis, fungal endophytes, abiotic stress tolerance, agriculture

## Abstract

Studies were undertaken to determine if fungal endophytes from plants in stressful habitats could be commercialized to generate climate resilient crop plants. Fungal endophytes were isolated from weedy rice plants and grasses from South Korea and the USA, respectively. Endophytes (*Curvularia brachyspora* and *Fusarium asiaticum*) from weedy rice plants from high salt or drought stressed habitats in South Korea conferred salt and drought stress tolerance to weedy rice and commercial varieties reflective of the habitats from which they were isolated. Fungal endophytes isolated from grasses in arid habitats of the USA were identified as *Trichoderma harzianum* and conferred drought and heat stress tolerance to monocots and eudicots. Two *T. harzianum* isolates were exposed to UV mutagenesis to derive strains resistant to fungicides in seed treatment plant protection packages. Three strains that collectively had resistance to commonly used fungicides were used for field testing. The three-strain mixture (ThSM3a) increased crop yields proportionally to the level of stress plants experienced with average yields up to 52% under high and 3–5% in low stress conditions. This study demonstrates fungal endophytes can be developed as viable commercial tools for rapidly generating climate resilient crops to enhance agricultural sustainability.

## 1. Introduction

Exacerbated by climate change [1], drought, increasing temperatures, and soil salinization threaten agricultural sustainability. Impacts of climate change are already decreasing agricultural productivity globally. For example, in Northern India, where the majority of farmers are small land holders without irrigation, monsoons are the only source of water and have become undependable [1]. Crop yields have suffered due to drought and high heat conditions that are now commonplace. In Asia and Africa, rice production has declined by 15–20% over the last 20 years due to increased temperatures, drought, and soil salinization [2,3,4,5]. Moreover, rice is particularly sensitive to nighttime temperatures above 24 °C. It is now common in many locations around the world where nighttime temperatures are reaching 29 °C during the growing season [2]. High temperatures impact spikelet anthesis and fertility, resulting in a decreased grain yield [6]. Elevated temperatures also increase the occurrence of rice diseases, resulting in significant economic losses [7,8,9].

By generating abiotic stress tolerant crops, the human impacts of climate change in agriculture could be lessened. For more than 40 years, three approaches have been taken to develop stress tolerant crops: genetic modification, mutational selection, and breeding traits from wild plants that have adapted to high stress habitats [10]. However, these efforts have had limited field success, presumably because stress tolerance involves genetically complex processes and the ecological/evolutionary mechanisms responsible for plant stress tolerance are still being defined [11,12].

All plants in natural ecosystems are thought to be symbiotic with fungal endophytes that reside within the plant [13]. These intimate associations play a pivotal role in the fitness and survival of plants thriving in stressful habitats. For example, fungal endophytes have been shown to adapt plants in coastal and geothermal habitats to salt and heat stress, respectively [14]. In addition, fungal endophytes can regulate plant growth, development, and reproduction [13,14,15,16,17,18]. Without their endophytes, plants do not survive in these habitats, indicating that stress adaptation involves symbiotic processes.

Weedy rice is widely distributed in rice-planting areas all over the world, particularly in South and South-east Asia, South and North America, and Southern Europe [19,20]. Weedy rice is classified as the same species as commercial rice varieties (*Oryza sativa*) and includes both major subspecies Indica and Japonica. In addition, it is common for weedy rice plants to exhibit tolerance to drought, cold temperatures, high salinity, and heavy metal resistance [21,22,23]. Because weedy rice is successfully acclimatized and adapted to thrive in habitats varying in soil types and environmental stresses, weedy rice is considered an important reservoir for genetic variation and has been implemented in breeding programs to improve commercial rice lines for enhanced traits of interest [22,24]. Indeed, many useful traits have been bred into commercial rice lines, such as enhanced blast disease-resistance, cold tolerance, and enhanced grain quality [20,21]. However, attempts to breed drought, salt, or heat tolerant rice lines have had minimal success [25].

The objective of this project was to determine if endophytes could be commercially developed and used to generate climate resilient crops capable of tolerating drought, heat, and salt stress. Fungal endophytes were isolated from three habitats imposing abiotic stress on resident plants: drought or salt stress on weedy rice in two locations in South Korea and drought and salt stress on resident plants in the USA. Although the weedy rice endophytes conferred sufficient stress tolerance, the fungal species are known as plant pathogens, making it difficult to obtain regulatory approval for agricultural use, and so were not pursued for commercial development. However, strains of endophytic *Trichoderma harzianum* isolated from native grasses in the USA were found to confer abiotic stress tolerance to multiple stresses and did not present a regulatory impediment. Due to the extensive use of fungicides on crop seeds, two *Trichoderma harzianum* isolates AST-TT08 and AST-TS10 were mutagenized with UV light to generate fungicide resistant strains. Three strains with resistance to different fungicides were mixed in equal proportions and applied as a seed treatment for field testing. The three-strain mixture was designated ThSM3a and was field tested on monocot and eudicot crops in North and South America, Australia, Europe, India, and Africa and commercialized as the microbial inoculant BioEnsure^®^. The most dramatic results were observed with poor small land-holding farmers in Rajasthan, India where it is commonly very hot and dry throughout the summer growing season. The endophytes significantly increased the yields of two staple food crops for the region, namely pearl millet and mung bean. We propose that this symbiotic technology can be used to mitigate impacts of climate change in agriculture and has the potential to break the chain of poverty for poor farmers globally.

## 2. Materials and Methods

### 2.1. Isolation and Identification of Endophytes from Weedy Rice

Endophyte isolation: Weedy rice seed lines containing their seed husks were collected (2008) from native arid (N = 7) and coastal (N = 8) habitats from observed high drought or moderate to high salinity (>6000 ppm NaCl, >13 mM NaCl, >5.9 g/L NaCl, EC > 12 dSm^−1^ NaCl) habitats of South Korea, respectively. Seeds were obtained from Dr. Hak Soo Suh, Wild Crop Germplasm Bank, Yeungnam University, Geongsan, South Korea. Seven drought tolerant weedy rice lines *Oryza sativa* L. were the Japonica type (2559, 2581, 2604, 2691, 2716, 2733, 2861), six salt tolerant lines (2518, 2582, 2687, 2692, 2693, 2844) were of the Japonica type, and two salt tolerant lines (22024, 22099) were the Indica type. One-hundred seeds with their seed husks of each rice line were surface sterilized by placing seeds in 0.5% (*v*/*v*) sodium hypochlorite for five minutes, rinsed in 70% ethanol for 15 s, rinsed with 5–10 volumes of sterile distilled water, and placed on 0.1× potato dextrose agar (1.5% agar) medium (PDA) supplemented with 100 μg/mL of ampicillin and chloramphenicol, and culturable fungi was allowed to grow out of samples at 25–28 °C under a 12 h light regime for 5–10 days. Fungal growth was observed out of the seed husks and the dominant endophytes (present in ≥70% of the seeds analyzed from each habitat) were selected and cultured, with single spores isolated and identified using classic microbiological and DNA sequencing techniques. The dominant endophytes present in weedy rice seeds from arid and high salt habitats was *Curvularia brachyspora,* and *Fusarium asiaticum,* respectively.

Fungal endophyte identification: Conidiophore and conidia morphology of the fungal isolates assigned them to the genus *Curvularia* and *Fusarium* [26,27,28]. Subsequent DNA sequence analysis of the variable ITS1 and ITS2 sequences of rDNA (described below) identified the species as *C. brachyspora,* and *F. asiaticum*.

DNA extraction, PCR amplification and sequence analysis: Prior to pigment formation, mycelia were harvested from fungi grown in 50 mL liquid cultures (1.0× potato dextrose broth supplemented with 100 μg/mL of ampicillin and chloramphenicol). Cultures were grown with agitation (150–180 rpm) at 25–28 °C with a 12 h fluorescent light regime. Mycelia were harvested by filtering cultures though four layers of sterile gauze and washing the mycelia mass with 3–5 volumes of sterile water. The mycelial mass was drained, and 0.1 g resuspended in 5 mL lysis buffer (150 mM EDTA, 50 mM Tris; pH 8, 2% Sarkosyl) and frozen at −80 °C. Once frozen, samples were exposed to 65 °C for five min and cell debris pelleted at 10,000 rpm for 15 min. Nucleic acids were precipitated from the supernatant with 0.7 volumes of PEG/NaCl (20% polyethylene glycol 8000, 2.5 M NaCl) for 10 min at 25–28 °C, DNA pelleted at 10,000 rpm for 15 min and resuspended in 0.4 mL 10 mM Tris (pH 8). DNA was precipitated with two volumes of 95% ethanol, NaCl to a final concentration of 0.1 M, and pelleted at 10,000 rpm for five min. The pellet was then resuspended in 100 µL of 10 mM Tris (pH 8) and DNA (0.5–2 µL) was used to PCR amplify ITS1, ITS2, and 5.8 s regions of the nuclear ribosomal DNA (nrDNA) repeat using the primer pair ITS1-F (5′-ggaagtaaaagtcgtaacaagg-3′) and ITS4 (5′-tcctccgcttattgatatgc-3′) [29]. PCR products were sequenced via Sanger sequencing at the University of Washington’s High Throughput Genomics Center. Sequences were analyzed and corrected using Sequencher^TM^ and aligned to annotated sequences in the NCBI BLAST nucleotide database.

### 2.2. Generation of Fungal Spores and Plants for Testing

Fungi: *C. brachyspora* and *F. asiaticum* were cultured on 0.1× potato dextrose agar (1.5% agar) medium (PDA) supplemented with 100 μg/mL of ampicillin and chloramphenicol, and fungal cultures grown at 25–28 °C with a 12 h fluorescent light regime. After 5–14 days of growth, conidia were harvested from plates by adding 10 mL of sterile water and gently scraping off conidia with a sterile glass slide. The final volume of conidia was adjusted to 100 mL with sterile water, filtered through four layers of sterile cotton cheesecloth gauze. Conidial suspensions were centrifuged at 6000 rpm for 10 min and resuspended in 100 mL sterile deionized water. The final spore suspension and concentration were adjusted to 10^3^–10^4^ conidia/mL depending upon the colonization assay chosen.

Plants: Rice seeds were immersed in 2.5% (*v*/*v*) sodium hypochlorite for 24–48 h, rinsed with 5–10 volumes of sterile distilled water, and imbibed in 10–20 volumes of water for 8–12 h. Seeds were germinated on 1% agar water medium plates, maintained at 28–30 °C and exposed to a 12 h fluorescent light regime for 1–2 days when seed germination was first observed to obtain known germinated seeds. Plant seedlings devoid of culturable fungal endophytes were obtained by growth of germinated seeds for an additional 1–2 days on plates. Studies began by choosing seedlings that showed no outgrowth of fungi into the surrounding medium.

### 2.3. Rice Seedling Growth Response

Plant growth response of weedy rice seedlings: Surface sterilized weedy rice seedlings of a salt (22024) and drought (2861) tolerant line were treated with *F. asiaticum* (SFa) and *C. brachyspora* (SCb), respectively, by immersion of germinated seedlings into conidial suspensions of 1000 conidia/mL in a plastic bag for 24 h at 28–30 °C with a 12 h fluorescent light regime to generate symbiotic (S) plants. Untreated control plants (UT) were mock inoculated by the immersion of seedlings into sterile distilled water. Plant seedlings were taken out of the conidial suspensions or distilled water and placed into sterile vials containing sterile water and exposed to a 12 h fluorescent light regime for five days at 28–30 °C and wet biomass (mg) weights measured and representative seedlings photographed to demonstrate growth response.

### 2.4. Magenta Box-Experiments of Weedy Rice Endophytes

Plant colonization: Surface-sterilized rice seedlings of weedy rice lines (see Section 2.2) a commercial USA variety (*Oryza sativa* cultivar M206, Japonica type) and a commercial South Korean variety (*Oryza sativa* cultivar Dongjin, Japonica type) were generated and transplanted into sterile double-decker magenta boxes (modified magenta boxes to hold soil or sand in upper chamber, and fluid in lower chamber that is wicked-up though a cotton rope [14]) containing equivalent amounts (380 +/−5 g) of sterile-sand such that nine magenta boxes with six plants/magenta box were produced (N = 54 rice seedlings/rice line or cultivar). The lower chamber was filled with 200 mL of sterile fluid (1× Hoagland’s solution supplemented with 5 mM CaCl_2_). The term double-decker magenta box and magenta box will be used interchangeably. After one week, plants were either mock inoculated with water (UT, untreated) or with fungal endophytes (S, symbiotic) by pipetting 1000 fungal conidia at the base of the crowns or stems and plants allowed to grow an additional week. At week two, one magenta box/treatment containing six 2-week-old rice seedlings was randomly selected to assess fungal colonization. Plant seedlings were surface sterilized by immersion into 0.5% (*v*/*v*) sodium hypochlorite for 20 min and rinsed with 5–10 volumes of sterile distilled water. The imprint technique was utilized to verify adequate surface sterilization of plant tissues was achieved [30]. Using aseptic techniques, plant tissues were cut into small sections (5 cm) representing the roots and shoots and placed onto 0.1× PDA (1.5% agar) medium supplemented with 100 μg/mL of ampicillin and chloramphenicol. Plates were incubated at 25–28 °C under a 12 h light regime for 7–10 days and assessed for the growth of culturable fungi out of plant tissues. Fungal species were identified using standard microbiological techniques to verify colonization occurred with the appropriate endophyte. Plants for laboratory studies were grown <2 months in double decker magenta boxes (four magenta boxes/treatment with six plants/magenta box = 24 plants) at 28–30 °C with a 12 h fluorescent light regime.

#### Abiotic Stress

Magenta box-experiments were performed with 2–4 week old plant seedlings. Magenta boxes were randomly moved every 2–3 days to mimic a randomized block design. Rice seedlings were generated that were either untreated (UT) or treated (S) with *C. brachyspora* (SCb) for drought, *F. asiaticum* (SFa) for salt stress, and both symbiotic treatments assessed for fluid consumption studies. Plants were assessed for health upon termination of the studies. Plant health was assessed on a 1–5 level with 1 being green and healthy, 2–3 indicating slight to moderate wilting, 4 indicating severe wilting and chlorosis, and 5 indicating wilted and dead. The images in the figures are representative of all replications of each treatment for the study presented.

Drought stress: Watering was terminated on 4-week-old UT and SCb plants by decanting off the fluid in the lower chamber of the double-decker magenta box and letting the plant and soils dry out over 10 days. A hydraProbe (Stevens-Vitel Inc., Chantilly, VA, USA) was used to ensure that soil moisture levels were equivalent between treatments when watering was terminated. After 10 days, plants showed symptoms (i.e., UT plants dead or severely wilted) and were re-hydrated by addition of 200 mL sterile water to the upper chamber of the magenta box. Plants were allowed 48 h to recover and assessed for health and photographed.

Salt stress: UT and S -Fa plants were exposed to 50–300 mM NaCl (EC = 5.9–26.0 ds/m) solutions (1× Hoagland’s solution supplemented with NaCl and 5 mM CaCl_2_; referred to as 50 mM, 100 mM, 150 mM, 200 mM and 300 mM NaCl solutions) in a step-wise manner. Two-week old seedlings were exposed to 50 mM NaCl solution for seven days, at which point the 3-week old plants were then exposed to 100 mM NaCl solution for seven days, followed by exposure to 150 mM and 200 mM NaCl solutions each for seven days, and a final exposure for two weeks to 300 mM NaCl. Salt solutions (200 mL) were filled in the lower chamber of the double-decker magenta boxes as needed. Upon termination of the magenta box studies, plants were assessed for health and photographed.

### 2.5. Mature Greenhouse Plant Studies

Plants for greenhouse studies were generated first in magenta boxes as described above. An additional 24 plants/treatment (four reps of six plants/pot) of UT, SCb and SFa plants were generated for weedy rice plant lines (N = 15) and the two commercial rice varieties Dongjin and M206. Two-week old rice seedlings were transplanted as a cluster of six plants into 7.5 L pots containing potting soil (Sunshine Mix#4) and grown at 28–30 °C to maturity (an additional 3.5 months) under paddy conditions (saturation of soil).

Drought stress: Seed yields and plant biomass (g) were assessed after four months with greenhouse grown UT and SCb treatments. Studies were conducted with seven weedy rice drought tolerant lines and two commercial rice varieties Dongjin and M206. Plants were grown for 13 weeks and watered with 1× Hoagland’s solution supplemented with 5 mM CaCl_2_. After 13 weeks, watering was terminated for ≤21 days to induce drought. All control plants were maintained at 28–30 °C and watered throughout the experiment with sterile water or 1×Hoagland’s solution supplemented with 5 mM CaCl_2_.

Salt stress: Greenhouse grown UT and SFa plants of eight weedy rice salt tolerant lines and two commercial rice varieties (Dongjin and M206) were assessed for salt stress tolerance. After sufficient growth was established, one-month old plants were exposed in a stepwise manner to increasing concentrations of salt solutions (1× Hoagland’s solution supplemented with 5 mM CaCl_2_ + 50 mM, 100 mM, 150 mM or 200 mM NaCl) every three weeks. Pots were flooded until soil was saturated with the various NaCl solutions to induce salt stress. Upon termination of the studies, plants were assessed for plant biomass and seed yields.

### 2.6. Fluid Consumption

Four-week-old plants grown in magenta boxes grown in the absence of abiotic stress were measured for the amount of fluid consumed by commercial rice varieties M206 and Dongjin treatments UT, SCb and SFa. Plants were grown (four magenta boxes/treatment with six plants/magenta box = 24 plants) at 28–30 °C with a 12 h fluorescent light regime. At week four, the bottom tier of the double-decker magenta box was removed and 200 mL of 1× Hoagland’s solution supplemented with 5 mM CaCl_2_ added. After 14 days, the remaining fluid was measured to determine fluid usage. Plants were grown at 28–30 °C under a 12 h fluorescent light regime.

### 2.7. Development of Symbiotic Technology for Commercialization

*Trichoderma harzianum* isolates AST-TT08 and AST-TS10 were obtained from bunchgrass species *Brachypodium sylvaticum* and *Muhlenbergia rigens*, respectively, thriving in moderate to high arid and saline soil stressed habitats in California, USA. The isolation and identification were done in the same manner as described for weedy rice endophytes (Section 2.1.) Identification of the conidiophore and conidial morphology of fungal isolates determined the genus as *Trichoderma* [26,27,28]. Subsequent PCR based sequence analysis of the variable ITS1 and ITS2 sequences of rDNA (described above) identified the species as *T. harzianum*. Conidia were produced by growing fungal cultures on 0.1× potato dextrose agar (1.5% agar) medium (PDA) supplemented with 100 μg/mL of ampicillin and chloramphenicol, and incubated at 25–28 °C with a 12 h light regime. After 7–10 days of growth, conidia were harvested from plates by adding 10 mL of sterile water and gently scraping off conidia with a sterile glass slide. The spore count was found >10^6^ conidia/100 mM petri plate. The final spore concentration was adjusted to 10^2^–10^8^ conidia/mL depending upon the assay chosen for the studies.

### 2.8. Generating Fungicide Resistant Strains of T. harzianum and Formulating for Field Testing

Conidia of *T. harzianum* strains were adjusted to 10^8^ conidia/mL, placed in a glass dish, and exposed to 0, 15, 30, 45, 60, and 90 s to UV light until 99.99% lethality was achieved. The UV light was placed 4–6 cm above the spore samples. After irradiation, samples were spread plated onto 0.1× PDA (1.5% agar) medium supplemented with 100 μg/mL of ampicillin and chloramphenicol in the absence or presence of various concentrations of fungicide chemicals commonly used in agriculture. The concentration for each fungicide used for selection was determined by spread plating wildtype conidia on petri plates containing various concentrations of each fungicide and identifying which concentration inhibited fungal growth completely. These levels equated to industry usage rates applied by seed treaters on a per seed basis. UV irradiated and non-irradiated *T. harzianum* AST-TT08 and AST-TS10 isolate conidia were spread plated on 100 mm media plates containing lethal levels of fungicides (hymexazol, ipconazole, prothioconazole, tebuconazole and thiabendazole). Plates (N = 3 per chemical tested) were incubated at 25–28 °C under a 12 h light regime for 7–10 days. Fungal colonies growing on chemical selection plates were considered to have high (H), moderate (M), or low (L) chemical resistance with 70–100%, 30–69%, and 0–29% conidial viability, respectively. Chemically resistant isolates were maintained for further testing. Phenotype stability on selection medium and plant colonization were criteria used to select candidates for field testing. Obtaining strains with resistance to multiple fungicides required 2–3 successive rounds of mutagenesis. For field testing, conidia from three strains that were collectively resistant to all of the commonly used fungicides were mixed (1:1:1) into a consortium designated ThSM3a.

### 2.9. Screening T. harzianum Strains for Biopesticidal Activity by Interaction Plating

These studies were performed by inoculating ThSM3a and another fungus onto opposite sides of 90 mm petri plates containing 0.1× PDA according to published protocols [31]. Antagonism was assessed between a mixture of the fungicide resistant strains (ThSM3a) and either *Aspergillus niger*, *Colletotrichum gloeosporioides*, *Colletotrichum magna*, *Curvularia protuberata*, *Curvularia inaequalis*, *Alternaria alternata*, *Fusarium culmorum*, *Penicillium,* or *Fusarium* spp. Plates were monitored visually for the growth of both fungi to determine if there were antagonistic interactions such as inhibition halos, excessive branching, or swelling of the mycelia hyphae as they grew close to one another. When the dual cultures grew within 1 cm of each other, the interaction zones were monitored microscopically. All assays were repeated three times.

### 2.10. Field Testing for the Evaluation of ThSM3a

Plants for field testing: Corn (*Zea mays*), barley (*Hordeum vulgare)*, soybean (*Glycine max*), rice (*Oryza sativa*), cotton (*Gossypium hirsutum*), pearl millet (*Cenchus americanus*, commonly known as the synonym *Pennisetum glaucum*) and mung bean (*Vigna radiata*) of various commercial varieties/hybrids were used for field testing in the USA, Argentina, Uruguay, Australia and India. All of the field trials were conducted by independent cooperators [Farmers, seed companies, agricultural cooperatives, universities, and contract research organizations (CRO’s)]. Standard seeding rates were used for crop planting as befitting the region and country. Seeds were treated with ThSM3a using standard industry rotary seed treaters in all countries with the exception in India, where small manual and electric rotary drums were fabricated for spray application of seeds. The application dose of ThSM3a was dependent upon the size of the seed. Large (corn, soy, cotton), medium (mung bean, rice, barley), and small (pearl millet) sized seeds were treated with conidia concentrations ranging from 2000–5000, 500–1500, and 50 conidia/seed, respectively. Specifics of the trials (year, locations, plot sizes, replications, treatment, cooperators) are listed in the figure legends. Trials and plots sizes varied from 10 ft × 50 ft to 50 acres with 1–8 replications/trial. All treated seeds regardless of their label designation in the field studies presented was treated with the ThSM3a formulation.

### 2.11. Screening ThSM3a for Biopesticidal Activity in Field Plots

All of the field testing for biopesticidal activity was performed by faculty at Michigan State University, Texas A&M AgriLife Research University and Oregon State University from 2013–2018 in locations with historical disease incidence. Testing was performed on soybean, rice, and barley. The field tests were comparative between ThTMS3a treated vs untreated control plants and the diseases screened were sudden death syndrome of soybean (*Fusarium virguliforme*), barley scald (*Rhyncosporium secalis*), and stripe rust (*Puccinia striiformis*) of barley, and brown spot of rice (*Cochliobolus miyabeanus*). In all of these field tests there was sufficient disease pressure to detect significant biopesticidal activity if present.

For soybean sudden death syndrome (SDS) screening, plots were randomly distributed throughout the field and soybean field plots were four rows at 76 cm spacing and 5.3 m long, planted on 1 May 2013 and harvested on 8 October 2013. There were 15–45 replicate plots/treatment, two treatments, control (untreated), and treated (3000 ThMS3a conidia/seed), and three soybean varieties tested. Yield data consists of percent moisture, kg/plot, Bushels/Ac, and the corrected Bushels/Ac. Some plots were damaged by the pivot irrigation. As a result, estimates of row feet lost were made and data corrected for the damaged sections. Stand counts were complete counts of one of the center two rows. Vigor rating 9 (bad) → 1 (good); an estimate of average plant vigor (score of 5) was determined and then plots were rated compared to that average.

Soybean SDS ratings were conducted on 16 August and SDS ratings done using a 0–9 scale (0 = no symptoms, 9 = dead). Disease severity (DS) ratings were done by estimating the average SDS value for affected plants and the disease incidence (DI) was estimated as the percentage of affected plants/plot. The disease index (DIX) was calculated using the equation ((DS/9)*DI) and used to compare plots. MSU staff collected and analyzed all the data.

For barley scald and stripe rust screening, barley plots were 37 m long (six plots, 6.1 m 1.5 m per plot, six rows per plot and 90 g seed per plot). Spatially, varieties were planted north/south in strips across the field. In the spring, plots are mowed and a 1.5 m alley between plots (east-west) created to facilitate access. Thus, the harvested area per plot is about 1.5 × 4.6 m. After measuring the actual plot length, the data was extrapolated to yield in kg/ha. Disease incidence was measured throughout the growing season and both resistant and susceptible varieties were treated (ThMS3a) for comparison to controls (untreated). Each year from 2015–2018, multiple barley varieties were tested with six replicate plots/treatment (untreated control and ThMS3a treated) and plots were randomly distributed in the field. Oregon State University staff collected all the data.

For rice brown spot screening, plots consisted of seven 4.6 m rows, and spaced 17.8 cm between rows. Three treatments [control, ThSM3a1 (500 conidia/seed) and ThMS3a2 (1500 conidia/seed)] were arranged in a randomized complete block design with four replications. Seeds of Presidio were treated with ThMS3a by AST staff prior to planting. Rice was drill seeded at 135 kg/ha on 11 May 2017. A permanent flood was established on 11 May to control weeds. Plots received 168 kg N/ha of Nature Safe (10-2-8, N-P-K) on 20 June. On 23 May, stand was counted as the number of seedlings in the three central rows of the plot and converted to stand per row foot. On 23 May, height of six seedlings randomly selected from each plot was measured. On 24 August, the severity of brown spot was rated on a scale of 0 to 9 where 0 = no symptoms and 9 = most severe damage (100% leaf area covered with lesions and most portion of leaves dead). Plots were not harvested at maturity for the determination of rice grain yield and milling quality due to the hurricane Harvey.

### 2.12. Screening ThSM3a for Soil Competitiveness and Presence in Plant Tissues

Soils: Samples were collected at the base of corn plants, between plants and between the rows (N = 10/location) in field test plots in Yakima, WA before planting, during cultivation and after over-wintering. The top debris layer (4–5 cm) was removed and a soil corer used to obtain <20 cm deep samples. Samples were placed into sealable plastic bags and stored at 4 °C until analyzed. Soils were homogenized and 100 g of soil of each sample retrieved for analysis using standard microbiological dilution plating methods. To begin, a teaspoon of measured soil was placed into 30 mL of sterile water and vigorously vortexed for 30 s, large soil debris allowed to settle for 5 s, and then serially diluted in a 1:10 dilution series seven times. Then, 0.3 mL sample of each dilution was spread plated onto a 150 mm, 0.1× PDA plate supplemented with 100 μg/mL of ampicillin and chloramphenicol. Dilution samples that exhibited approximately 100 CFU/plate was chosen as the target dilution for feasible CFU counts and fungal species identification. The target dilution was then plated 10 more times onto 0.1× PDA (1.5% agar) medium supplemented with 100 μg/mL of ampicillin and chloramphenicol without (non-selection media) and with chemical fungicides (selection media) and incubated at 25–28 °C under a 12 h light regime for 7–10 days. Collectively, these 10 replicate plates equated to approximately 1000 fungal CFU per soil sample from untreated and ThMS3a treated plants (soils collected from the base of corn plants, between plants, and between the rows, one month before, during (two weeks, two- and three-months post-planting), and after the growing season (four months post-harvest)) and were assessed for the presence of *Trichoderma* spp. The chemical fungicides chosen to visualize the potential presence of ThMS3a in soils was thiabendazole, hymexazol and tebuconazole, applied at lethal levels to the medium such that sensitive wildtype isolates could not grow, but fungicide resistant Trichoderma isolates could. Growth of Trichoderma on selection media plates were further analyzed microscopically to verify they were *T. harzianum*.

Plant residue: One-year post harvest of the Yakima plots, ThSM3a treated and untreated corn plant residue (crowns and upper roots) were collected from the two 6 ha plots to determine if the endophytes had survived over winter. Samples (N = 10) were collected from the center of each plot, cleaned with water, either surface sterilized or not. Plant samples were surface sterilized by immersion into 0.5% (*v*/*v*) sodium hypochlorite for 20 min and rinsed with 5–10 volumes of sterile distilled water. The imprint technique was utilized to verify adequate surface sterilization of plant tissues was achieved [30]. Using aseptic techniques, plant tissues were cut into small sections (5 cm) representing the roots and shoots and placed onto non-selection or selection media plates (see above). Plates were incubated at 25–28 °C under a 12 h light regime for 7–10 days and assessed for growth of culturable fungi out of plant tissues. Fungal species were identified using standard microbiological techniques.

### 2.13. ThSM3a Field Trials

Rice, barley, soybean, corn, pearl millet, cotton, and mung bean seeds were treated using rotary treaters commonly used in the agricultural community. Depending upon the crop, 50–5000 conidia/seed of the *T. harzianum* three-strain mix ThSM3a were applied one week to nine months before planting. Seeds were planted in fields using manual methods or standard commercial equipment under standard conditions. Plot sizes ranged from four rows × 3 m to 20 ha and commercial seed varieties with standard plant protection packages (PPP’s) were used for testing. All testing was comparative between ThSM3a treated and untreated control seeds and field trials were carried out by farmers, seed companies, agricultural distributors, university faculty, agricultural cooperatives and contract research organizations (CRO’s). More details about the field trials are included in the figure legends.

### 2.14. Quantifying Abiotic Stress

Quantifying abiotic stress relies on a myriad of factors that are nearly impossible to completely capture. In our effort to assign stress levels, we created a simple ranking system based on six common and quantifiable abiotic stressors: number of growing season days above 32 °C (heat stress), level of irrigation utilization (drought stress), soil composition, precipitation, plant growth phase of endophyte application, and plant health. Each is defined and described below. High abiotic stress at the location and year of a trial is represented with a high combined stress score. Lower abiotic stress is represented by a lower combined stress score. Stress rankings in the 24 trials ranged from 3–35. Stress definitions and ranking methodology:

(1) Number of growing season days above 32 °C (heat stress): Using data from the National Climatic Data Center (NCDC) and the National Oceanic and Atmospheric Administration (NOAA), totals for the number of days during the growing season (April–September for US, June–November for India) that exceeded 32 °C were recorded at each location. Ranks were then given to each location; 1 was assigned to 0–10 days, 2 to 11–20, etc. for a maximum rank of 19.
(2) Irrigation levels (drought stress): Ranks were determined based off the amount of irrigation provided at each location. If no irrigation was utilized a rank of 10 was given. If a trial was 100% irrigated a rank of 0 was given. Partial irrigation scores were determined on a case by case basis and scored either 5 or 2.5 depending on irrigation conditions at each site.
(3) Soil composition: Using location specific data and definitions from the United States Department of Agriculture (USDA) Web Soil Survey, hydrologic soil groups were categorized based off estimates of runoff potential and overall total sand composition was determined. Soils falling into group A (high infiltration rate when thoroughly wet) were regarded as higher stress soils. Soils with high group A percentages were ranked with higher scores whereas soils with loams or clays were ranked lower. Starting with 0% sand composition with a ranking of 0, one point was added for every 5% increase in sand composition resulting in a total of 10 points for highly stressed soil.
(4) Precipitation (drought stress): Using historical climate data from NOAA, departures in precipitation from 30-year averages were determined at each location. If precipitation for that year and locale was above the 30-year average a 0 was given as a stress value. For every inch of precipitation below the 30-year average one point was given, rounding to the nearest whole number (range 0–12).
(5) Plant growth phase at time of application: Ranking ranged from 0–3 based on application onto seeds (0), seedlings (1), multi-leaved vegetative plants (2), and reproductive plants (3). The intent is that the earlier the application, the better stress protection.
(6) Plant health at time of application: Ranking is based on symptoms of stress impact on plants at the time of endophyte application. To decrease potential for subjective ranking bias, this was simplified to a three-ranking system: 0 for healthy plants lacking any sign of stress, 5 for significant leaf curling or browning, and 10 for severe wilting.

### 2.15. Statistical Analyses

Due to the variety and type of data collected, several methods of statistical analysis were performed throughout this paper as indicated in the table captions and figure legends. For all experiments unless otherwise stated, the student’s t-test was used to determine significance between two groups (i.e., untreated control vs. treated). A significance level of *p* < 0.05 was considered to be statistically significant unless otherwise specified.

## 3. Results

### 3.1. Weedy Rice Endophytes

**Figure 1:**
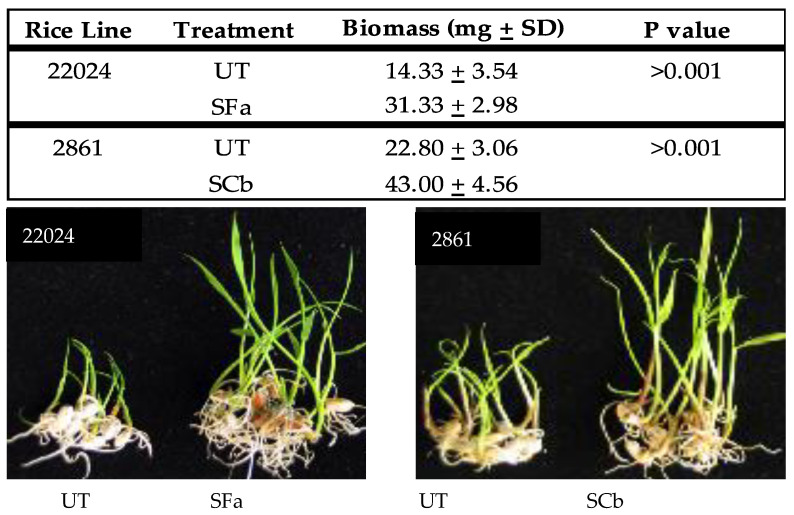
Weedy Rice Seedling Growth Response. Statistical analysis (N = 6) of seedling wet biomass weights (mg) with the seeds removed, was performed and S treatments found to be significantly larger (*t* test). Representative photos (bottom panels) showing growth response differences of five day old (N = 6) weedy rice seedlings lines 22024 (salt tolerant) and 2861 (drought tolerant) without (untreated = UT) or with (symbiotic = S) their native endophytes *C. brachyspora* (SCb) and *F. asiaticum* (SFa), respectively.

The vegetative tissue surrounding the seeds (husks) of weedy rice lines collected from drought and high salt stress natural habitats were analyzed for the presence of endophytes. Two dominant endophytes (present >70% of samples) were identified using morphological traits and DNA sequence analysis. Drought resistant lines had *Curvularia brachyspora* in 71% of the samples and salt tolerant lines had *Fusarium asiaticum* in 87% of seeds analyzed. In order to determine if these were mutualistic fungal endophytes, sterile weedy rice and commercial rice seedlings were generated to test for the ability of these endophytes to colonize seedlings and promote detectable levels of initial growth response, a trait often observed with endophytes [17]. Both *C. brachyspora* (SCb) and *F. asiaticum* (SFa) imparted a growth response that was statistically significant when compare to untreated (UT) plants (Figure 1).

**Figure 2:**
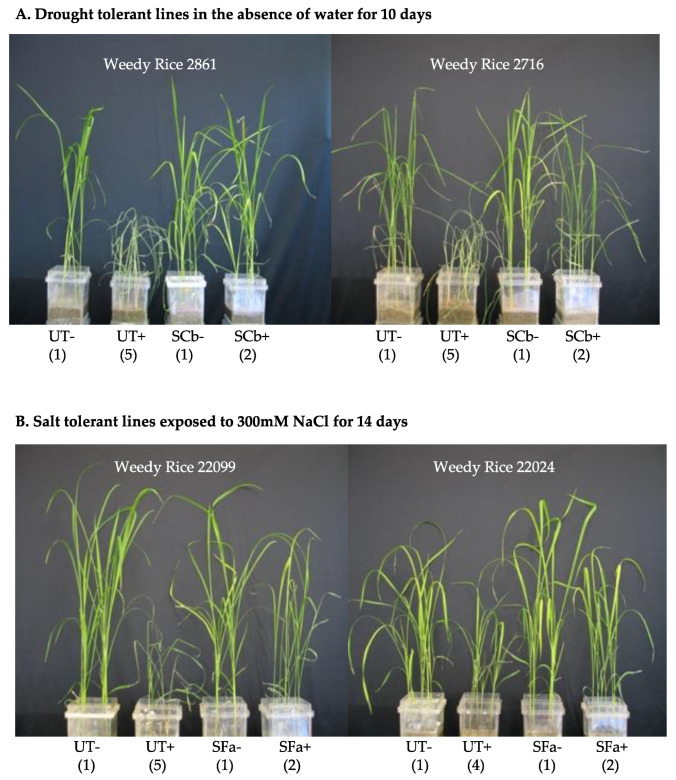
Drought and Salt Responses of Weedy Rice Lines. Untreated (UT) and symbiotic (S) plants were generated with native endophytes isolated from weedy rice lines from drought (SCb; *C. brachyspora*) or salt stress (SFa; *F. asiaticum*) habitats. Representative photo of magenta box studies showing: (**A**) Drought tolerance performed on one-month old plants of weedy rice drought tolerant plant lines 2861 (panel A-left) and 2716 (panel A-right) in the absence (−) and presence (+) of drought stress (devoid of water for 10 days) of UT (untreated) and symbiotic plants (SCb); (**B**) Salt tolerance assays were performed on 2-week old weedy rice drought tolerant plant lines 22099 (panel B-left) and 22024 (panel B-right) in the absence (−) and presence (+) of salt stress (exposure to 300 mM NaCl for 14 days) of UT (untreated) and symbiotic plants (SFa). Assays were conducted with six plants/magenta box and 4 magenta boxes per treatment. Plant health was numerically scored collectively for the six plants in each magenta box. The Kruskal–Wallis as a non-parametric test was utilized as the plant health metric is categorical data. Plant health is represented by the numbers under the plants. Regardless of the stress, S plants out performed their UT counterparts (Weedy Rice 2861 H(1) = 7, *p* = 0.008151; Weedy Rice 2716 H(1) = 6.4, *p* = 0.01141; Weedy Rice 22099 H(1) = 7, *p* = 0.008151; Weedy Rice 22024 H(1) = 6.22, *p* = 0.01262). No significant differences in plant health was observed with treatments in the absence of stress.

To determine if endophytes from weedy rice conferred stress tolerance, sterile rice seedlings were grown in double decker magenta boxes and three experimental groups generated: untreated (UT) and symbiotic plants containing either *C. brachyspora* (SCb) or *F. asiaticum* (SFa). Plants were exposed to drought or salt stress and plant health assessed. SCb plants remained healthier and showed lower levels of drought stress symptoms for longer periods of time when compared to UT plants (Figure 2A). SFa plants that were exposed to <300 mM NaCl for two weeks showed fewer symptoms of salt stress compared to UT plants (Figure 2B). These results indicated that both dominant endophytes from weedy rice imparted stress tolerances that were reflective of the habitats from which they were isolated. Representative photos showing drought and salt tolerance conferred by *C. brachyspora* (SCb) or *F. asiaticum* (SFa) are presented in Figure 2A,B.

To determine if the abiotic stress tolerance observed was unique to the weedy rice-fungal associations, both endophytes were tested for conferring drought and salt stress tolerance to weedy rice lines (N = 17) and commercial varieties Dongjin and M206. *C. brachyspora* and *F. asiaticum* conferred drought and salt stress tolerance, respectively, to both weedy rice and commercial varieties tested in laboratory seedlings (Figure 2) and greenhouse mature plants taken to seed set (Table 1). Symbiotic (S) and untreated (UT) weedy rice lines and commercial varieties were grown to maturity and assessed for biomass and seed yield (Table 1). Endophyte colonized plants performed significantly better than UT plants under both drought and salt stress.

**Figure 3:**
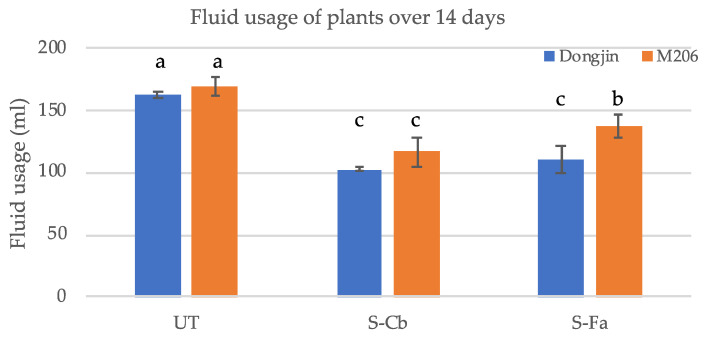
Fluid Consumption in Untreated and Treated Plants. Fluid consumption was measured on four-week old UT (untreated) and symbiotic (with SCb or SFa endophytes from weedy rice lines from drought and salt stress habitats, respectively) Dongjin (blue bars) or M206 (orange bars) rice plant varieties grown in double decker magenta boxes over a 14-day period. Statistical analysis indicated that S plants consumed less fluid compared to UT plants with S-Dongjin cultivar consuming the least amount of fluid (Duncan’s Multiple Range Test, *p* < 0.001). Different letters above the bars indicate statistically significant differences.

Fluid usage efficiency is another trait often associated with fungal endophytes [14]. One-month old symbiotic (S) and untreated (UT) Dongjin and M206 plants were grown in double decker magenta boxes to assess water usage. Both SCb and SFa plants showed significantly lower fluid usage when compared to UT plants of both Dongjin and M206 indicating that symbiotic plants consumed less fluid than UT plants (Figure 3). General observations showed that, upon exposure to abiotic stress, the overall plant health of endophyte colonized weedy rice lines and commercial cultivars were superior compared to UT plants for both drought and salt stress. The appearance of drought stress symptoms over a 10-day period occurred up to four days later in SCb plants compared to UT plants (Figure 2). The overall plant survival was 50–75% higher in SFa plants compared to UT plants (not shown) exposed to high salinity (<21 days 200 mM NaCl). The majority of symbiotic plants developed greater biomass (5–204%) and grain yields (11–114.5%) than UT plants (Table 1). The exceptions were symbiotic weedy rice line 2861 and M206 under drought stress, and M206 under salt stress showed no significant biomass differences compared to UT plants. Weedy rice 2259, a drought tolerant line, and salt tolerant 2582 and 2693 lines did not show differences in seed yields between S and UT treatments. The seed yields of weedy rice drought tolerant 2604 and salt tolerant 2518 were not determined due to insect herbivory and/or microbial pathogen impacts.

### 3.2. Development of Symbiotic Technology for Agriculture

#### 3.2.1. *Trichoderma harzianum* Strains for Field Testing

To avoid complications with using weedy rice fungal endophytes for commercialization (isolates of these species are known to be plant pathogens), we focused on *Trichoderma harzianum* endophytic strains isolated from annual grasses from an arid region in CA, USA. Since commercial crop seeds are commonly coated with fungicides, it was necessary to determine the sensitivity of the *T. harzianum* strains to fungicides commonly applied as part of seed treatment packages. While the strains had inherent resistance to some of the fungicides, they were inhibited by several of the commonly used fungicides. To overcome fungicide sensitivity, conidia were mutagenized with UV light and selected for resistance. Fungicide resistant strains were isolated for each fungicide category and tested for resistance to the respective fungicides (Table 2). Three strains that collectively had resistance to conazoles, thiabendazole, and hymexazol, were then mixed for field testing. The three-strain mixture (ThSM3a) asymptomatically colonized the roots of both monocots and eudicots. Colonization occurred following the development of appressoria on plant tissues and strains were recovered after surface sterilization of plant tissues (not shown).

#### 3.2.2. Assessing Soil Competence of ThSM3a

Since the *T. harzianum* strains in ThSM3a were not found in rhizosphere soils of the original plants (not shown), it was of interest to determine if they expressed biopesticidal activity and were capable of growing in field soils. Rhizosphere and soil competence of ThSM3a was tested in corn field plots (two plots approximately 6 ha each) in Yakima, WA, USA by collecting soil samples approximately one month before and two weeks after planting, during the mid (two months) to late (three months) stages of the growing season, and a final collection after harvest (four months post-harvest) during the winter. Samples were plated on selective (with fungicides) and non-selective (without fungicides) media. The first sampling indicated that the ThSM3a strains were not present in the rhizosphere soils. Sampling two weeks after planting showed the ThSM3a strains were present in all of the rhizosphere soils from treated plants (Table 3), but not present in soils between plants, in furrows (not shown) or associated with untreated plants (Table 3). The strains were not detected in any soils collected at 2–3 months after planting or four months post-harvest. Not surprisingly, control soil samples that were plated on media without fungicides, revealed low levels of *Trichoderma* spp. in all samples (>10%) and the majority (<90%) being other fungal species commonly found in soils (*Aspergillus*, *Fusarium*, *Alternaria*, *Mucor*, *Penicillium*, *Curvularia*, and *Colletotrichum* spp.) for all time points. However, further analysis on selective media showed that the *Trichoderma* spp. from untreated control plots were not resistant to conazoles, thiabendazole or hymexazol, whereas Trichoderma isolates from ThMS3a treated soils did show resistance to these chemicals and was identified though microscopy as *T. harzianum* (Table 3). Although there may be Trichoderma isolates that have resistance to one of these chemicals, resistance to all three is more unlikely.

It was of interest if ThMS3a could over winter and survive in residual plant tissues. One-year post harvest of the Yakima plots, ThSM3a treated and untreated corn plant residue was collected. The crown and upper roots were chosen as this is where the fungi reside in high density in growing plants. Ten of each plant tissue type was collected from the two plots, and were either surface sterilized or not and placed on non-selection and selective media containing fungicides to determine if ThSM3a could survive in plant tissues. None of the samples had fungicide resistant ThSM3a strains emerge. Samples that were not surface sterilized and plated on non-selective media did have different fungal species emerge, but not Trichoderma species, and no fungal growth observed on selection media (Table 3).

#### 3.2.3. Assessing Biopesticidal Activity of ThSM3a

Since *Trichoderma harzianum* has a reputation of having biopesticidal activity, dual plating interaction and microscopic analyses were performed with ThSM3a against five plant pathogenic fungi and four non-pathogenic fungi (Table 4). The ThSM3a strains did not inhibit the colonial or mycelial growth of the other fungi. However, the colonial growth of the ThSM3a strains was inhibited by several fungi (*Aspergillus niger*, *Colletotrichum gloeosporioides*, *Alternaria alternata*, *Fusarium culmorum*, *Fusarium* spp.). The only mycelia impact observed was by ThSM3a, which had increased branching in the presence of the plant pathogen *Colletotrichum gloeosporioides* (Table 4).

ThSM3a was further evaluated for biopesticidal activity against several fungal pathogens under field conditions. All of the field testing was performed by faculty at Michigan State University, Texas A&M AgriLife Research University and Oregon State University. The diseases screened were sudden death syndrome (SDS) of soybean (*Fusarium solani*), barley scald (*Rhyncosporium secalis*) and brown spot on rice (*Cochliobolus miyabeanus*).

Biopesticidal activity against Sudden Death Syndrome (SDS) of Soybean was performed by Michigan State University on 60 randomly distributed plots (four rows at 76 cm spacing and 5.3 m long). Average Disease Index for Sudden Death Syndrome (SDS) of Soybean showed no significant differences between treatments (*t*-test, *p* value = 0.38, not shown).

**Figure 4:**
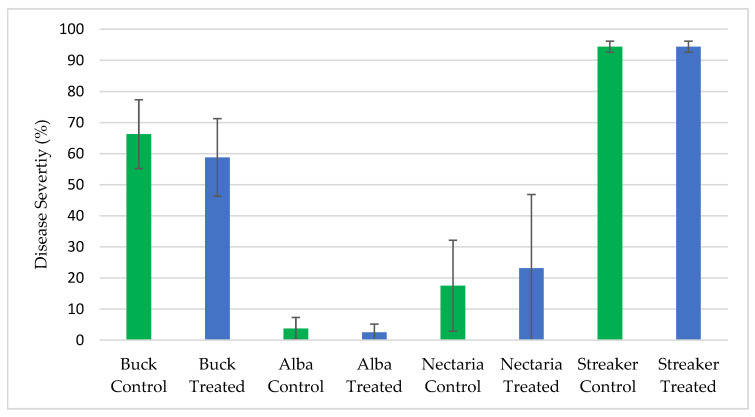
Percent Barley Scald by Location and Treatment. Untreated controls (green bars) and ThSM3a treated (blue bars) barley of four varieties were tested. Each treatment had six replicate plots for a total of 48 plots. Plots were randomly distributed in the field. The higher the percentage, the higher the disease incidence. There were no significant differences between treatments (ANOVA *p*-value = 0.986 (F = 0.000281 < Fc = 4.0195)). The single factor ANOVA test is the most appropriate for looking at the differences between means when the dependent variable is a percentage. Trials performed by Oregon State University.

**Figure 5:**
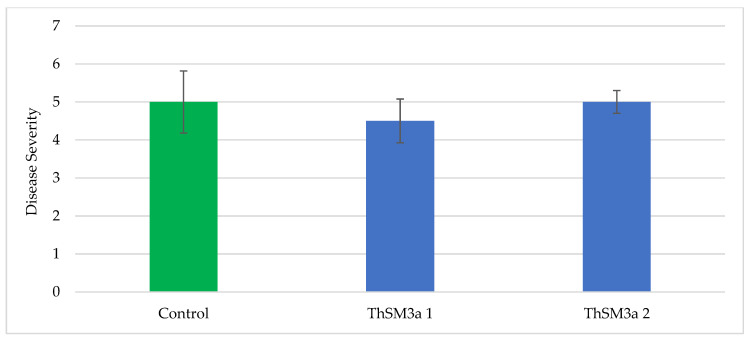
Average Severity of Brown Spot on Rice by Treatment. Brown spot symptoms were rated on a scale of 0–9 (the higher the number, the higher the disease severity) and the Kruskal-Wallis test determined there was no significance. H(3) = 2.0556, *p* = 0.3578. Untreated control is indicated by the green bar. The blue bars are ThSM3a treatments 1 and 2 which represent low (500 CFU/seed) and high dose (1500 CFU/seed), respectively. Plots were randomly distributed throughout the field. Plots consisted of seven 4.6 m rows, and spaced 17.8 cm between rows and replicated four times per treatment. Kruskal–Wallis as a non-parametric test was utilized as the symptom variable is categorical data. Trials performed by Texas A&M AgriLife Research.

Additional statistical analysis showed there were no significant differences between ThSM3a treated and untreated crops with regard to barley scald (Figure 4) and brown spot on rice (Figure 5).

#### 3.2.4. Field Assessment of ThSM3a

Field testing of ThSM3a began in 2012 and expanded every year both geographically and with the number of crops tested. Field tests were performed and harvested by independent organizations, universities, companies or farmers. Twenty-five crop species were field tested in the USA, Argentina, Australia, Uruguay, and India. Positive beneficial responses in the form of biomass, stress tolerance, and yields were observed in all crops (Table 5).

**Figure 6:**
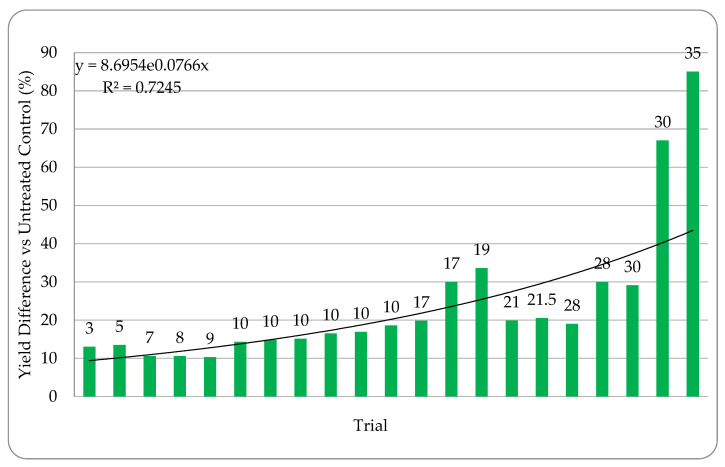
Corn Percent Yield Difference vs Stress. Stress increases left to right from 3–35 with level of stress indicated above each bar as described in the materials and methods. Higher stress levels are indicated by higher numbers. Trials were performed in CA, WA, MI, NE, MN, AZ, ND, and India using 10 commercial hybrids with standard PPP’s. Each bar is a separate trial and plots sizes varied from 10 ft × 50 ft to 50 acres. The bars indicate the percent difference in yields of ThMS3a treated versus the untreated controls. Each test had a single replication of treated and untreated so it was not possible to include standard deviations or statistical analysis. Cooperators: CRO’s, seed companies, farmers, universities.

**Figure 7:**
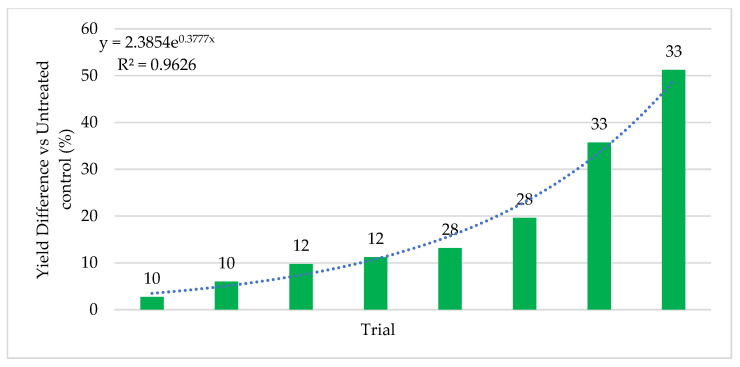
Cotton Percent Yield Difference vs Stress. Stress increases left to right from 10–33 with level of stress indicated above each bar as described in the materials and methods. Higher stress levels are indicated by higher numbers. Trials were performed in TX, Australia, and India using four commercial hybrids with Standard PPP’s. Each bar is a separate trial and plots sizes varied from 10 ft × 50 ft to 50 acres. The bars indicate the percent difference in yields of ThMS3a treated versus the untreated control. With increasing stress, increases in yields were observed. Each test had a single replication of treated and untreated so it was not possible to include standard deviations or statistical analysis. Cooperators: CRO’s, seed companies and farmers.

Field tests comparing ThSM3a treated and untreated plants were performed to assess the response of a monocot (corn, Figure 6) and a eudicot (cotton, Figure 7) to increased levels of heat and/or water stress. The field plots ranged in size from four rows × 12.2 m to 4 ha and stress was assigned based on six factors: (1) the number of days above 32 °C, (2) irrigation or dry land cultivation, (3) soil composition, (4) precipitation, (5) plant growth phase and time of treatment, and (6) plant health and time of treatment. ThSM3a increased corn yields between 5% and 85%, reflective of stress levels. The average yield increase was 26% and the win rate was 93% (tests with yield increases above 5%).

Similar results were observed with cotton (Figure 7). ThSM3a increased cotton yields on average 18% with a range of 2.7–51% and a win rate of 87% (tests with yield increases above 5%).

**Figure 8:**
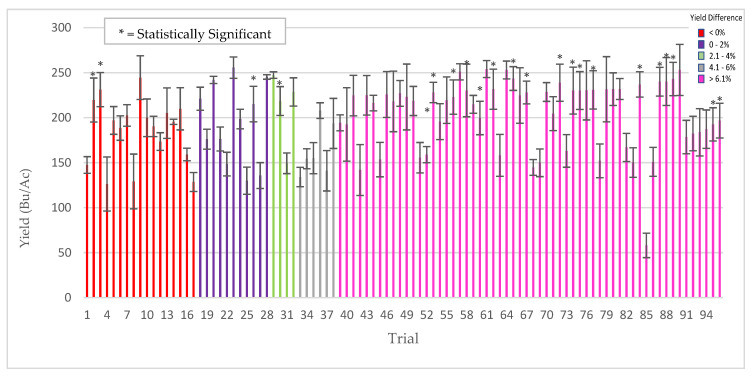
Corn response to low stress conditions. From 2013–2017, 96 trials were performed in 17 US states using 35 commercial varieties with standard PPP’s. Each bar is a separate trial comparing yields of treated (ThMS3a) vs untreated plants. Plots sizes varied from 10 ft × 50 ft to 5 acres with 4–8 replications/trial. Bars are color coded according to yield differences (Bu/Ac) indicated in the legend (upper right of graph). Bars with an asterisk symbol (*) indicate yield significance (*t* test, *p*-value < 0.05). Cooperators: seed companies, farmers, CRO’s, universities, Ag distributors.

To demonstrate the impact of abiotic stress on this symbiotic association, comparative studies were done with corn, wheat, and alfalfa to assess ThSM3a benefits under low levels of stress during cultivation. The results with corn were dramatically different with an average yield increase of 3.5% (+323 kg/ha) and a win rate of 69% (tests with yield increases above 1%) (Figure 8).

**Figure 9:**
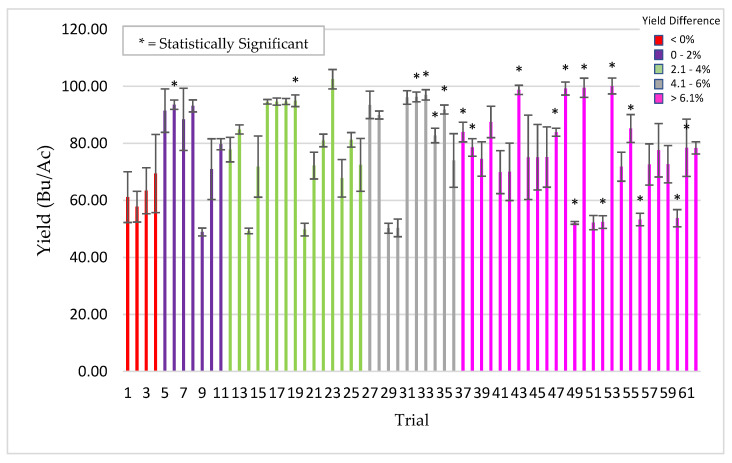
Winter wheat yield response (treated plants vs untreated controls) under low stress conditions. From 2017–2020, 62 trials were performed in three US states with commercial varieties/hybrids with Standard PPP’s. Each bar is a separate trial comparing yields of treated (ThMS3a) vs untreated plants with 4–8 replicates/trial. Plot sizes varied from 10 ft × 30 ft to 10 ft × 50 ft. Bars are color coded according to yield differences (Bu/Ac) indicated in the legend (upper right of graph). Bars with an asterisk symbol (*) indicate yield significance (*t* test, *p*-value < 0.05). Cooperators: South Dakota State University, Agri-Tech Research, seed company.

Similar results were observed in winter wheat with an average yield increase of 5.3% and a win rate of 82% (tests with yield increases above 2%) (Figure 9).

**Figure 10:**
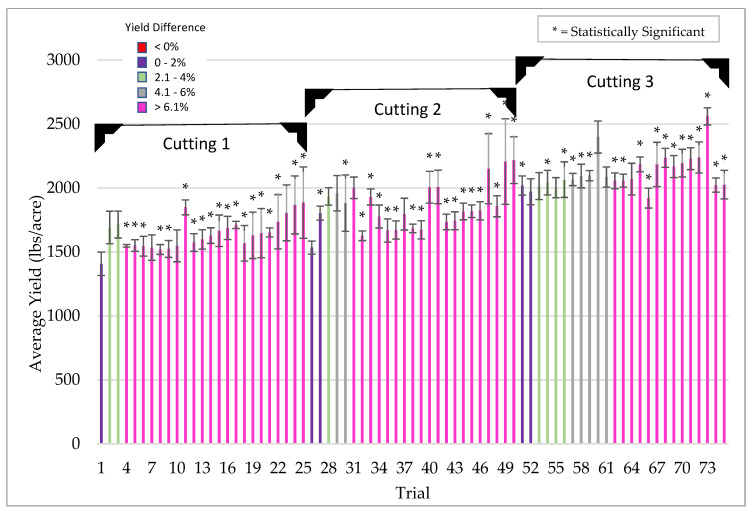
Alfalfa yield response of treated (ThMS3a) vs untreated controls plants under low stress conditions. From 2016–2020, 75 trials were performed in four US states (WI, SD, NV, AZ) on commercial varieties with standard PPP’s. Each bar is a separate trial and plots sizes varied from 10 ft × 50 ft to 10 acres with 4–8 replications/trial. In each trial, there were three successive cuttings every 1–1.5 months and is indicated as the average yield (lbs/acre). Bars are color coded according to yield differences indicated in the legend (upper left of graph). Bars with an asterisk symbol (*) indicate yield significance (*t* test, *p*-value < 0.05). Cooperators: farmers, CRO’s, universities.

Under low stress, ThSM3a increased yields of alfalfa an average of 12.7% with a win rate of 85% (tests with yield increases above 5%) (Figure 10).

#### 3.2.5. Field Assessment of ThSM3a in Rajasthan, India

**Figure 11:**
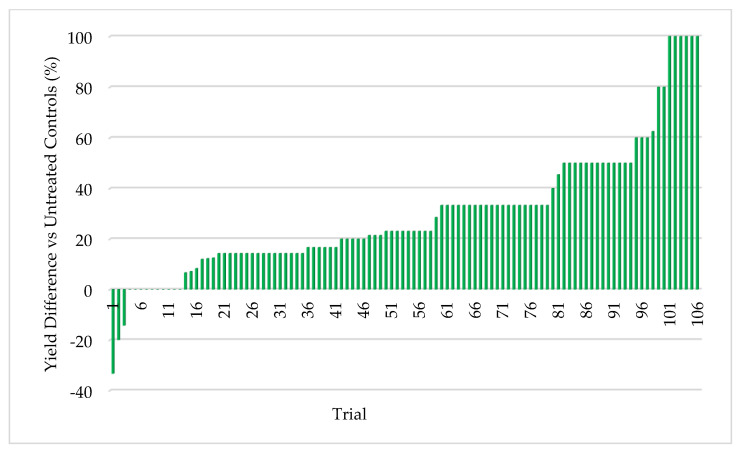
Pearl millet response to BioEnsure. 106 trials (0.1–5.0 acres/plot) were performed with carry-over and market seed. Each bar represents a separate comparative trial of treated (ThMS3a) vs untreated plant yields. Win rate was 96% with a minimum of 10% yield increase. Each test had a single replication of treated and untreated so it was not possible to include standard deviations or statistical analysis. Cooperators: farmers.

**Figure 12:**
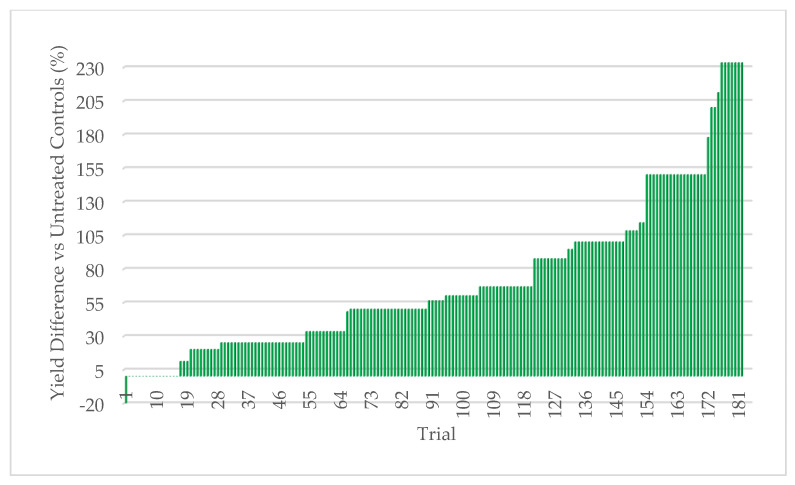
Mung bean response to BioEnsure. 183 trials (0.1–5.0 acres/plot) were performed with carry-over and market seed. Each bar represents a separate comparative trial of treated (ThMS3a) vs untreated plant yields. Win rate was 93% with a minimum of 10% yield increase. Each test had a single replication of treated and untreated so it was not possible to include standard deviations or statistical analysis. Cooperators: farmers.

AST was awarded a USAID/SWFF grant to accelerate the development of and bring endophyte technology to small land-holding farmers in India. The three-strain mixture (ThSM3a) was the basis of the product used in India commercially branded as BioEnsure. Rajasthan was chosen for testing because it has been experiencing weak monsoon rains for a decade and growing conditions are routinely above 37 °C. In 2016 and 2017, AST treated seeds of two staple crops, pearl millet (monocot) and mung bean (eudicot), for 400 farmers in 12 villages. The farmers were interested in anything that could impart heat and drought stress protection, and improve performance of carryover seed from previous crops. All of the trials were comparative between endophyte treated and untreated seeds and crops were grown by dry-land cultivation. The results exceeded expectations with the endophyte treatment increasing the average yields of pearl millet by 29% (Figure 11) and mung bean by 52% (Figure 12). Yield increases ranged from −33% to 100% for pearl millet and −20% to 230% for mung bean. The zero differences for both crops reflect direct comparisons between treated carryover seed (from a previous season) and untreated fresh market seed. There was no obvious explanation for the few negative yield results.

## 4. Discussion

Studies with weedy rice lines add to the body of knowledge that fungal endophytes play a significant role in the ecology, biology, and adaptation of plants [13,32,33,34]. It may also explain why breeding certain traits from native plants into domesticated plants has had limited success. Both *C. brachyspora* and *F. asiaticum* endophytes conferred stress tolerance, increased biomass, and decreased water consumption in weedy rice and other commercial rice cultivars, did not grow into seeds or embryos and were categorized as Class 2 endophytes [13]. Although endophytes can establish on seed coats of native plants, we have not observed endophytes establish on the seed coats of agricultural plants (not shown). Therefore, the endophytes that are responsible for abiotic stress tolerance are vertically transmitted inefficiently during the breeding process. This is supported by the fact that attempts to breed salt, heat, and drought tolerance from weedy rice into commercial varieties have been marginally successful in the field [35,36,37].

There are three basic requirements of commercializing endophytes for agricultural use: (1) the ability to scale production to meet demand, (2) field efficacy, and (3) regulatory approval [38,39]. Unfortunately, some isolates of *C. brachyspora* and *F. asiaticum* are known to be pathogenic. This presented a significant impediment to obtaining regulatory approvals so the isolates were not field tested or commercialized. Instead, a series of endophytes were screened for meeting all three requirements. Most intriguing were the endophytic strains of *Trichoderma harzianum*.

One impediment to field testing of fungal endophytes is the ubiquitous use of plant protection packages (PPPs) containing fungicides in commercial seed treatments of non-organic seed. Although the endophytic *T. harzianum* strains were resistant to several fungicides in PPPs, they were inhibited by others such as the sterol biosynthesis inhibitors (SBIs). Generating SBI-resistant strains required successive rounds of UV light mutagenesis and selection for resistance to one or more SBIs using plate inhibition assays. A group of three isolates (ThSM3a) that collectively showed resistance to all of the commonly used fungicides were used for field testing. There are numerous reports describing the benefits of fungal endophytes on seed germination, seedling vigor, abiotic, and biotic stress tolerance, and increased biomass of crop plants [38,40,41,42,43,44,45]. Although there are comparatively few reports on endophytes improving crop production under field conditions [38], fungal endophytes have shown good field efficacy for enhancing yields, biological control, and improving yield quality [38,43,44,45]. Extensive field testing demonstrated that ThSM3a conferred tolerance to drought and temperature extremes in both a monocot (corn) and eudicot (cotton). There was a strong relationship between the level of stress and yield benefits (greater stress = greater yield). Quantifying stress levels was complicated by inaccuracies between regional versus local environmental data including precipitation, soil composition, and temperature. This could be improved by increasing the accuracy of environmental data with on-site weather stations and field soil compositional analysis. Regardless, the six-factor system used for quantifying stress levels was sufficient to visualize the relationship between stress and yield benefit.

Since *T. harzianum* is commonly known to express biopesticidal activities and is a competitive soil species, ThSM3a was screened for both of these characteristics. Soil competitiveness was assessed by monitoring the presence of fungicide resistant *T. harzianum* in soil before and after planting ThSM3a treated and untreated corn seeds. Although there were *Trichoderma* spp. and other fungi in the soils before planting, no fungi grew on growth medium containing fungicides that ThSM3a could tolerate. Two weeks after planting seeds, fungicide resistant *T. harzianum* was isolated from rhizosphere soils but not in any of the subsequent samples collected up to one year after planting. The results suggest that ThSM3a is similar to other Class 2 endophytes in regard to soil competitiveness [13].

The biopesticidal potential of ThSM3a was assessed under laboratory and field conditions. Interaction plating of ThSM3a against nine fungi including seven plant pathogens demonstrated a lack of biopesticidal activity against any of the fungi. Field studies carried out to assess biopesticidal activity against sudden death syndrome of soybean (*Fusarium solani*), barley scald (*Rhyncosporium secalis*) and brown spot on rice (*Cochliobolus miyabeanus*) also revealed no biopesticidal activity by ThSM3a. The lack of biopesticidal activity and soil competitiveness is particularly interesting because mycoparasitism is considered an ancestral trait of the genus *Trichoderma* which is why so many strains have been commercialized as biopesticides [46]. Biopesticidal activity can occur via antagonism, hyperparasitism, or induced resistance depending on the species and strains [47]. Although *Trichoderma* spp. are known to colonize the outer regions of roots, a few species are known to be true endophytes living inside plant tissues. Interestingly, most of the endophytic species are thought to have recently diverged and are phylogenetically distinct from other *Trichoderma* spp. with biopesticidal activities [48].

To the best of our knowledge, this is the first assessment of the relationship between the level of abiotic stress and the resulting yield enhancement from fungal endophytes that confer stress tolerance. The strong relationship between increased stress levels and increased yields in corn (monocot) and cotton (eudicot) suggests that the symbiotic communication responsible for abiotic stress tolerance is conserved and predates the divergence of the plant lineages more than 130 million years ago [49,50,51]. The fossil record indicates that the relationship between plants and fungi are ancient and may have existed when plants colonized land during the Devonian geologic period [52]. It has been suggested that the ability of fungal endophytes to confer drought and heat stress may be been instrumental in the movement of plants onto land [51,52,53].

Symbiotic yield benefits were also observed in corn, wheat, and alfalfa cultivated under low-stress conditions. However, the yield increases were lower (5%) for corn than those observed under more stressful conditions (26%). Although wheat and alfalfa were not tested under high stress conditions, average yields under low stress were 5.3% and 12.7%, respectively. Moreover, the yield benefits in alfalfa were observed with each successive cutting in a single season. This also indicates that endophyte yield benefits occur in leguminous and non-leguminous plants.

As part of a USAID/SWFF grant, Adaptive Symbiotic Technologies was asked to bring endophyte technology to small land-holding farmers in climate-vulnerable regions of India. We chose to work in the state of Rajasthan, one of the hottest and driest regions in India where the majority of its 6.3 million farmers live on less than $5/day and cultivate on less than two hectares of land. The vast majority of crops are cultivated under dry land conditions with temperatures typically exceeding 40 °C during the growing season. There are two growing seasons: the monsoon season when planting occurs in June or July depending on rainfall and a fall/winter season if there is sufficient moisture in the soil for seed germination. Farmers grow crops for sustenance, fodder for animals, carryover seed, and revenues. If they do not produce sufficient yield for sustenance a famine ensues and the government provides wheat to rural communities.

The three-strain mixture ThSM3a was the basis of the product used in India commercially branded as BioEnsure. Although the intent of field testing was to compare yields from endophyte treated seeds to untreated seeds, many farmers had other issues they wanted to address. The primary concern was that the majority of farmers use carryover seeds from previous harvests because they cannot afford to purchase fresh market seed which costs 4–8 times more than the value of carryover seed. The famers lack proper seed storage facilities so the carry over seeds have lower percent germination, seedling vigor, and final yields compared to market seeds. Compared to untreated plants, the farmers observed that treated plants had increased percent germination, seedling vigor, plant biomass, stress tolerance, and yields. More biomass translated to increased fodder for animals that produce milk, their primary source of protein. Increased yields equate to more grain for sustenance and potential revenues. Most importantly for the farmers, treated carryover seeds performed at least as well or better than market seeds (https://vimeo.com/192003746, accessed on 20 April 2021).

As climate change has increased in severity, agricultural land around the world has become marginal for crop production, resulting in decreased food security [54]. Food insecurity plays a critical role in human migration, poverty, famine, and political unrest [55,56]. Unfortunately, there is a strong relationship between poverty, food insecurity, and political instability. This has been evident in Somalia, Syria, Honduras, and Venezuela where human migrations directly correlate with food insecurity. It is just a matter of time before food insecurity begins to impact more nations. Although there are physical and chemical technologies available that aid in water delivery, retention in soil, or adsorption in plant tissues, there are very few technologies that can generate climate resilient crops. Since the vast majority of farming around the world involves dryland cultivation, food security has become dependent on generating climate resilient crops. The symbiotic technology described here allows for the generation of climate resilient crops within a single growing season that can be applied to a diversity of monocots and eudicots. These studies demonstrate that symbiotic technology may be developed as a viable commercial tool for rapidly generating climate resilient crops to enhance agricultural sustainability.

## Figures and Tables

**Table 1:** Greenhouse drought and salt stress tolerance in mature weedy rice lines and commercial cultivars.

	**Biomass (g)**		**Seed Yields (g)**	

A. Drought Stress Weedy Rice Lines (N = 7)	S-*Cb*	UT	S-*Cb*	UT
	(SD ± 2.8–30.0)	(SD ± 4.5–20.1)	(SD ± 0.7–2.8)	(SD ± 1.0–3.7)
2559	456.7 ^a^	404.3 ^b^	24.6 ^a^	21.9 ^a^
2581	573.5 ^a^	510.0 ^b^	25.1 ^a^	11.7 ^b^
2604	56.2 ^a^	40.25 ^b^	ND	ND
2691	105.5 ^a^	34.7 ^b^	8.2 ^a^	0 ^b^
2716	580.2 ^a^	424.5 ^b^	35.5 ^a^	24.3 ^b^
2733	403.7 ^a^	330.3 ^b^	27.3 ^a^	20.4 ^b^
2861	389.5 ^a^	370.3 ^a^	22.7 ^a^	16.5 ^b^
Commercial Rice Cultivars (N = 2)				
Dongjin	192.7^a^	143.2^a^	11.6^a^	6.2 ^b^
M206	213.4^a^	181.9^a^	20.1^a^	12.8 ^b^
	**Biomass (g)**		**Seed Yields (g)**	

B. Salt Stress Weedy Rice Lines (N = 8)	S-*Fa*	UT	S-*Fa*	UT
	(SD ± 7.0–26.4)	(SD ± 3.7–28.1)	(SD ± 1.0–2.6)	(SD ± 0.8–2.6)
2518	132.2 ^a^	97.5 ^b^	ND	ND
2582	420.0 ^a^	360.8 ^b^	33.6 ^a^	30.2 ^a^
2687	530.2 ^a^	365.3 ^b^	49.5 ^a^	40.4 ^b^
2692	489.5 ^a^	393.5 ^b^	36.0 ^a^	29.5 ^b^
2693	483.5 ^a^	449.5 ^a^	38.7 ^a^	36.7 ^a^
2844	477.0 ^a^	405.5 ^b^	35.4 ^a^	20.8 ^b^
22024	481.0 ^a^	425.2 ^b^	46.4 ^a^	41.4 ^b^
22099	492.7 ^a^	446. 3^b^	31.2 ^a^	24.8 ^b^
Commercial Rice Cultivars (N = 2)				
Dongjin	120.4 ^a^	88.7 ^b^	9.6 ^a^	5.9 ^b^
M206	154.8 ^a^	137.3 ^a^	14.7 ^a^	8.3 ^b^

Drought and salt stress on mature rice plants (<4 months) under greenhouse conditions. Untreated (UT) and symbiotic (S) plants were generated with native endophytes isolated from weedy rice lines from drought (SCb) or salt stress (SFa) habitats. Six plants were grown in pots and ×4 pots/treatment (N = 24 total) for each rice line and cultivar tested. Seven drought tolerant weedy rice and two commercial rice varieties were grown for three months and drought stress imposed by termination of watering for 14 days. For salt stress, eight salt tolerant weedy rice lines and two commercial rice varieties were exposed to increasing salt solutions of 50, 100, 150 and 200 mM NaCl every three weeks. Plant biomass and seed yields were assessed upon termination of the studies. The seed yields of weedy rice drought tolerant 2604 and salt tolerant 2518 lines were not determined due to insect and/or microbial pathogen impacts. Statistical analysis was performed using Duncan’s Multiple Range test. Different letters and color shading indicate significant differences (*p* < 0.01). ND = Not Determined.

**Table 2:** Fungicide Compatibility of ThSM3a.

	Wildtype Strains	UV Irradiated Strains
Fungicides	Compatibility	Compatibility
Captan	H	NSF
Carbamide	H	NSF
Fludioxonil	M	NSF
Hymexazol	L	H
Ipconazole	L	H
Mefenoxam	H	NSF
Metalaxyl	H	NSF
Myclobutanil	M	NSF
Prothioconazole	L	H
Sedaxane	H	NSF
Strobulins	M	NSF
Tebuconazole	L	H
Thiabendazole	L	H
Thiophanate	H	NSF
Thiram	M	NSF

Compatibility levels: H = high; M = medium; L=low. NSF = Not selected for since wildtypes were already compatible. Fungicide levels that equated to industry usage rates applied by seed treaters on a per seed basis was added to chemical media selection plates (N = 3 per chemical). Fungal colonies growing on chemical selection plates were considered to have high (H), moderate (M) or low (L) chemical resistance based on conidia colony growth of 70–100%, 30–69%, and 0–29%, respectively, compared with growth in the absence of fungicides.

**Table 3:** Yakima soil analysis for the detection of fungicide resistant *T. harzianum* (ThTMS3a).

Sampling Time	Field Plot	Sample Analyzed	Non-Selection Media		Selection Media		
			-Fungicides		+Fungicides		
			Trich. spp.	Other Fungi	Trich. spp.	Other Fungi	*T. harzianum*
Pre-planting 1 month	Untreated	soil	+	++	-	-	-
	ThMS3a	soil	+	++	-	-	-
Post-planting 2 weeks	Untreated	soil at plant base	+	++	-	-	-
	ThMS3a	soil at plant base	+	++	+	-	+
Post-planting 2 and 3 months	Untreated	soil at plant base	+	++	-	-	-
	ThMS3a	soil at plant base	+	++	-	-	-
Post-harvest 4 months	Untreated	soil at plant base	+	++	-	-	-
	ThMS3a	soil at plant base	+	++	-	-	-
	Untreated	non-sterilized roots and crowns	-	+	-	-	-
	ThMS3a	non-sterilized roots and crowns	-	+	-	-	-
1 year post-harvest	Untreated	surface sterilized roots and crowns	-	-	-	-	-
	ThMS3a	surface sterilized roots and crowns	-	-	-	-	-

Two plots (6 ha) adjacent to each other were chosen for field studies to assess for the presence of ThTMS3a in soils and plant tissues throughout the year. One month prior to planting, soils were randomly assayed in 10 locations per plot. 20 cm soil core samples surrounding the plant root rhizosphere (N = 10) were collected at one-month pre-planting, 2 weeks, 2- and 3-months post-planting, and a final soil sampling 4 months post-harvest. Collectively, 10 non-selection (0.1 × PDA plate supplemented with 100 μg/mL of ampicillin and chloramphenicol) and selection media plates (media containing thiabendazole, hymexazol, and tebuconazole) representing <1000 fungal CFU were assayed for soil samples of each time point. Non-selection plates revealed >10% of the fungal CFU as *Trichoderma* spp. (Trich. spp.) indicating its presence in low numbers (+); compared to collectively the high presence (++; <90%) of other common soil fungi (*Aspergillus, Fusarium, Alternaria, Mucor, Penicillium, Curvularia*, and *Colletotrichum* spp.). A low level (+, >10%) of *Trichoderma* spp. isolates microscopically verified as *T. harzianum* were detected from rhizosphere soils from the base of plants collected 2 weeks post-plating. No other fungi were detected on selection media for any other soil samples. One-year post-harvest, plant crown and upper roots (N = 10) were collected from untreated and ThMS3a treated plots. Tissues were surface sterilized or not, and six 5 cm tissue samples representative of the roots and crowns for each sample, placed on non-selection and selection media plates. Growth of Trichoderma was not observed in any of the samples but other fungal species were present in soils and grew out of non-sterilized plant tissues plated on non-selection plates. The samples were scored for the presence (+) or absence (−) of fungi. CFU was not able to be determined as growth occurred out of plant tissues. Colors represent different treatments and organisms isolated from samples.

**Table 4:** Dual plate Interaction of ThSM3a and other fungi.

Fungi	Lifestyle	Plate Interaction	Microscopic
*Aspergillus niger*	saprophyte	TGI	NR
*Colletotrichum gloeosporioides*	pathogen	TGI	TMB
*Colletorichum magna*	pathogen	NI	NR
*Curvularia protuberata*	mutualist	NI	NR
*Curvularia ineaqualis*	mutualist	NI	NR
*Alternaria alternata*	pathogen	TGI	NR
*Fusarium culmorum*	pathogen	TGI	NR
*Penicillium* spp.	saprophyte	NI	NR
*Fusarium* spp.	pathogen	TGI	NR

Fungal Plate interactions: ThSM3a and another saprophytic, mutualistic, or pathogenic fungus (Aspergillus niger, Colletotrichum gloeosporioides, Colletotrichum magna, Curvularia protuberata, Curvularia inaequalis, Alternaria alternata, Fusarium culmorum, Penicillium, or Fusarium spp.) were inoculated onto opposite sides of a 0.1× PDA media plate and fungi allowed to grow within 1 cm of each other and microscopically assessed for the type of interactions observed. The interactions were scored as antagonistic when areas of growth inhibition or branching of mycelia was observed as a result of fungal interaction. When growth was not inhibited and no deviations in mycelia growth observed, these were considered non-inhibitory response. All assays were repeated three times and no standard deviations observed in the interactions. Abbreviations: TGI, growth inhibition of ThSM3a by other fungal species; TGM, mycelial branching of ThSM3a in response to other fungal species; NI, no inhibition of ThSM3a or the other fungal species; NR, no response by ThSM3a or the other fungal species.

**Table 5:** Crop Responses to ThSM3a.

Crop	Biomass	Stress Tolerance	Yield	Conditions
alfalfa	+	+	+	Field
barley	+	NYD	+	Field
blueberries	+	NYD	NYD	Greenhouse
canola	+	+	NYD	Field
carrots	+	+	+	Field
corn	+	+	+	Field
cotton	+	+	+	Field
cucumber	+	NYD	NYD	Greenhouse
dry beans	+	NYD	NYD	Field
field peas	+	+	+	Field
guar	+	+	+	Field
leafy greens	+	NYD	NYD	Greenhouse
millet	+	+	+	Field
mung bean	+	+	+	Field
okra	+	+	+	Field
onion	+	+	+	Field
pasture grass	+	+	+	Field
potato	+	NYD	+	Field
rice	+	+	+	Field
sesame	+	+	+	Field
sorghum	+	+	+	Field
soybean	+	+	+	Field
sunflower	+	+	+	Field
wheat (spring)	+	+	+	Field
wheat (winter)	+	+	+	Field

NYD, not yet determined. +, Positive response for increased biomass, stress tolerance or yield.

## Data Availability

The data presented in this study are available on request from the corresponding author.
